# Targeted provider education and pre-visit planning increase rates of formal depression screening in childhood-onset SLE

**DOI:** 10.1186/s12969-021-00576-4

**Published:** 2021-08-03

**Authors:** Evan Mulvihill, Rebecca Furru, Alana Goldstein-Leever, Kyla Driest, Stephanie Lemle, Darby MacDonald, Emily Frost, Vidya Sivaraman

**Affiliations:** 1 Department of Pediatrics, Divisions of a Rheumatology, Nationwide Children’s Hospital and The Ohio State University, Ohio Columbus, USA; 2 Department of Social Work, Nationwide Children’s Hospital, The Ohio State University, Ohio Columbus, USA; 3 Department of Psychiatry and Behavioral Health, Nationwide Children’s Hospital and The Ohio State University, Ohio Columbus, USA; 4 Department of QI Services, Nationwide Children’s Hospital and The Ohio State University, Ohio Columbus, USA; 5 Department of Pediatrics, Division of Rheumatology, Nemours/A.I. duPont Hospital for Children, and Thomas Jefferson University, Delaware Wilmington, USA; 6 Department of Pediatrics, Division of Rheumatology, Nemours Hospital for Children Division of Rheumatology, 1600 Rockland Road, DE 19803 Wilmington, USA

**Keywords:** Lupus, Mental health, Depression screening, PHQ-9

## Abstract

**Background:**

Despite being at high risk for depression, patients with childhood-onset systemic lupus erythematosus (c-SLE) are infrequently and inconsistently screened for depression by their pediatric rheumatologists. We aimed to systematically increase rates of formal depression screening for c-SLE patients in an academic Pediatric Rheumatology clinic.

**Methods:**

Our multi-disciplinary quality improvement (QI) team used electronic health record (EHR) documentation to retroactively calculate baseline rates of documented depression screening using the Patient Health Questionnaire-9 (PHQ-9). We then engaged key stakeholders to develop a clinical workflow for formal depression screening in the clinic. We also provided education to providers regarding mental health disorders in c-SLE, with an emphasis on prevalence, screening methods, and management of positive screens. We then used the Plan-Do-Study Act (PDSA) method of QI to systematically evaluate and adjust our process in real time. The primary outcome was the percentage of patients with c-SLE seen per month who had a documented PHQ-9 screening within the past year.

**Results:**

The percentage of children with documented PHQ-9 results ranged from 0 to 4.5 % at baseline to 91.0 % within 12 months of project initiation. By the end of the project, monthly screening rates greater than 80 % has been sustained for 10 months. As a result of these efforts, twenty-seven (48.2 %) patients with at least mild depressive symptoms were identified while seven (12.5 %) with thoughts of self-harm were referred to appropriate mental health resources.

**Conclusions:**

Routine formal depression screening is feasible in a busy subspecialty clinic. Using QI methods, rates of formal depression screening among children with c-SLE were increased from an average of 3.3 % per month to a sustained monthly rate of greater than 80 %. Individuals with depressive symptoms and/or thoughts of self-harm were identified and referred to appropriate mental health resources.

## Background

Childhood-onset systemic lupus erythematosus (c-SLE) is a chronic multi-systemic autoimmune disease with a prevalence of 3.3–24 per 100,000 children.[1] Compared to healthy children, those affected by c-SLE have 2.9 times increased odds of being diagnosed with depression and 5.4 times increased odds of endorsing suicidal ideation.[2] In one cohort, 47 % of young adults with SLE experienced at least one major depressive episode over a twelve-year follow up period.[3] A diagnosis of c-SLE has been shown to be an independent risk factor for depression, regardless of disease activity and duration. [3] Physical manifestations of the disease, including rashes and alopecia, as well as side effects associated with pharmacologic therapies, especially high-dose prednisone, are also predictive of incident depression in individuals with SLE.[4] These issues are compounded by systemic racism in the United States which results in social and health inequities that predominantly affect Hispanics and African Americans, two populations that are also disproportionately affected by SLE. [5] Inequities such as increased financial strain and lower educational attainment have both been directly linked to depression in patients with SLE. [6] Depression in SLE has been directly associated with medication non-adherence, resulting in objective measures of increased disease activity as well as worse patient reported outcomes in multiple domains. [3, 7]

 In general, children with c-SLE and their parents place equal emphasis on physical and emotional health.[8] While parents tend to feel comfortable discussing mental health issues with their child’s rheumatologist, children often withhold such concerns for various reasons including perceived social stigma, fear or uncertainty about interventions, potential for parental “emotional burden”, and concern for minimization of the issues by their physician.[8] Low rates of mental healthcare in patients with c-SLE are also likely affected by low rates of routine depression screening both by general pediatricians and pediatric rheumatologists. For example, only 2 % of surveyed pediatric rheumatologists reported that they perform routine depression screening for their patients despite 77 % agreeing on its importance.[9] These low screening rates exist despite recommendations from both the American Academy of Pediatrics (AAP) and the United States Preventive Services Task Force (USPSTF) that routine depression screening be performed annually for all children 12 years or older.[10, 11] Similar recommendations have been put forth by some disease-specific organizations such as the Cystic Fibrosis Foundation (CFF) and the European CF Society (ECFS).[12].

In August 2017, a multi-disciplinary quality improvement team was established in the Division of Rheumatology at Nationwide Children’s Hospital (NCH). This team initiated a quality improvement project with the specific aim of increasing rates of annual formal depression screening in patients with c-SLE in the Rheumatology clinic at NCH from approximately 3.3 %% to 80 % by August 2018. Once achieved, we aimed to sustain that rate for at least 6 months.

## Methods

### Context

NCH is a 476-bed, pediatric, quaternary care, academic medical center in the mid-western United States. The interdisciplinary rheumatology team consists of 7 pediatric rheumatologists, 2 nurse practitioners, nurses, a social worker, a clinical psychologist, a clinical pharmacist, and a quality improvement (QI) data specialist. Patients with c-SLE are evaluated either in the general Rheumatology clinic or in the multi-disciplinary “Lupus Clinic”. The multi-disciplinary clinic includes providers from Rheumatology, Nephrology, Pharmacy, Social Work, Psychology, and Neuropsychology. This QI project was piloted in the “Lupus Clinic” and later expanded to include all eligible patients with c-SLE who were seen in the NCH Rheumatology clinic. The Patient Health Questionnaire 9 (PHQ-9), a validated nine item patient-administered questionnaire, was chosen for its ease of use, its ability to measure depression symptom severity, its proven reliability and validity for use among adolescents, and its known acceptability among pediatric patients with lupus and their parents.[13–15] An electronic PHQ-9 flowsheet in the EHR allowed for convenient documentation and review of responses to individual questions as well as total score. This flowsheet existed in the institution’s EHR prior to initiation of the project but had not been widely used.

### Quality Improvement (QI) team

**Fig. 1:**
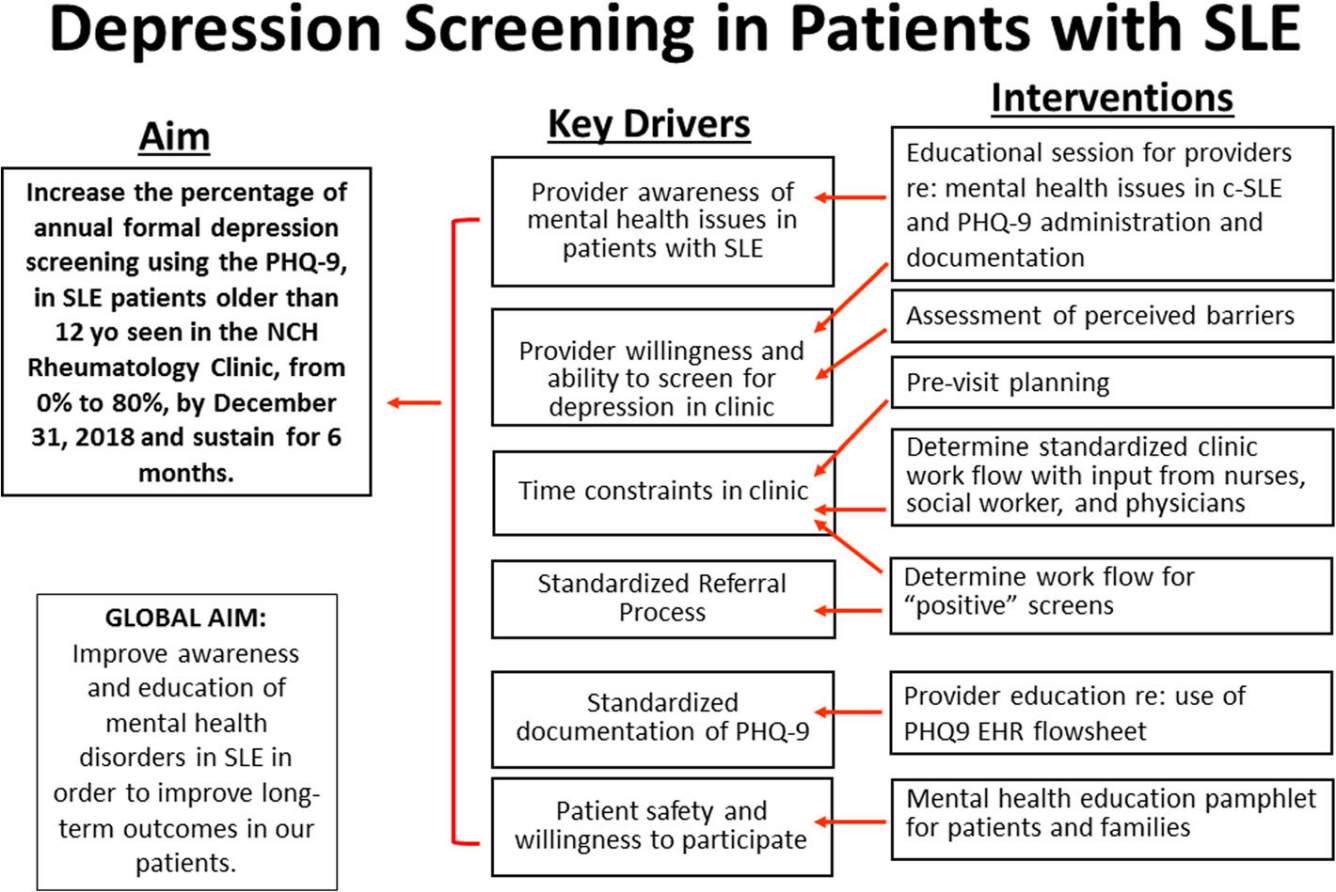
Key Driver Diagram

The QI team consisted of pediatric rheumatologists, a clinical social worker, and a clinical psychologist. Input and feedback was intermittently solicited from Rheumatology clinic staff and Rheumatology nurses. Based on a survey of provider habits and perceived barriers performed prior to introduction of the study, the QI team developed a key driver diagram (Fig. 1) to identify major factors impeding routine formal screening of patients with c-SLE for depression in the Rheumatology clinic. One key driver identified to increase screening rates included increased provider education regarding mental health disorders in c-SLE, with an emphasis on prevalence, screening methods, and management of positive screens. A streamlined process for screening, documentation, and referral was also important to the success of the project, as was patient and parent buy-in.

### Study population

All Rheumatology physicians and nurse practitioners were included in calculation of monthly screening rates. However, patients with c-SLE were primarily seen by physicians. Patients were included in the baseline rate calculation if they had received medical care in the Pediatric Rheumatology clinic at NCH from January 2017 to July 2017. After initiation of the project, patients were eligible to be screened with the PHQ-9 and included in the project if they had a new or prior diagnosis of c-SLE based on ICD-10 codes listed in the electronic health record (EHR). Eligible participants were also required to be at least 12 years of age at the time of their visit, in keeping with published recommendations set forth by the AAP, USPSTF, CFF, and ECFS.[9–11] Patients who were not English-speaking or who had documented developmental delay were excluded from formal screening for the purposes of this project. The project was initially piloted in the multi-disciplinary “Lupus Clinic” and quickly broadened to include all patients with a c-SLE diagnosis receiving medical care in the Rheumatology clinic at NCH.

### Baseline formal depression screening rates

From January 2017 to August 2017, baseline rates of formal PHQ-9 depression screening were collected manually from the PHQ-9 EHR flowsheet. These baseline screening rates preceded the establishment of the Lupus Clinic and ranged from 0 to 4.5 % each month. The average baseline screening rate per month was 3.3 %.

### Interventions

Our first intervention aimed to increase the awareness of mental health disorders in patients with c-SLE among Rheumatology providers. During a Rheumatology division meeting, data on the prevalence of depression in SLE and the low rates of formal screening for depression among pediatric rheumatologists nationally were presented. Results from a survey of NCH provider habits performed prior to the meeting were also reviewed. Finally, the plan to implement a QI project was discussed and feedback from providers was solicited.

**Fig. 2:**
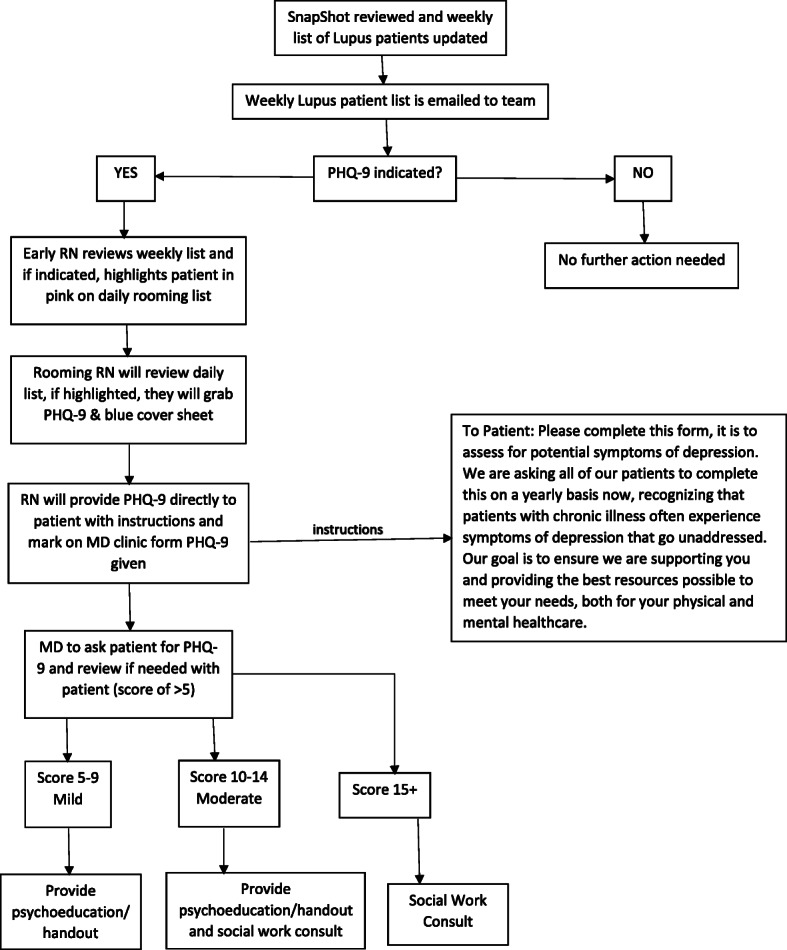
Clinic Workflow. *Snapshot refers to list of all patient scheduled for visits in the Rheumatology clinic. **Early RN refers to nurse who comes in to prepare the clinic before arrival of the first patient. ***Rooming RN refers to the nurse who leads a patient and family to their assigned room

**Fig. 3:**
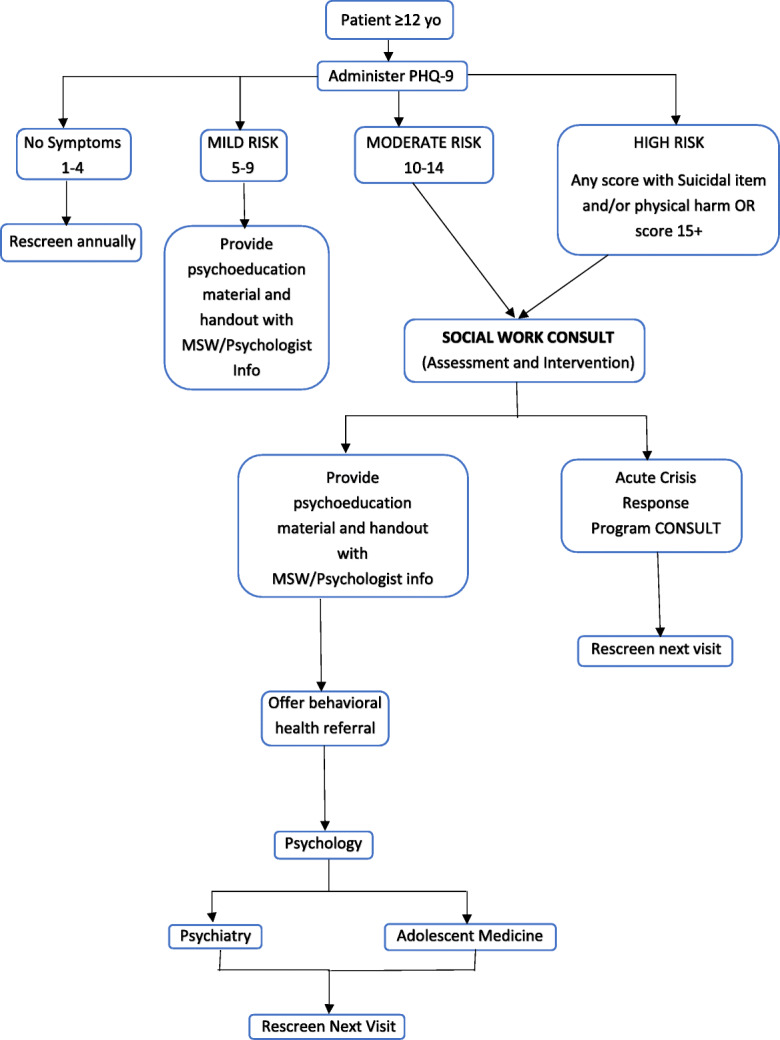
PHQ-9 Referral Flowsheet

The second intervention emphasized the importance of a streamlined clinic workflow. Initially, our licensed social worker reviewed patients scheduled to be seen in Lupus Clinic on a weekly basis. As a member of the Lupus Clinic, she was present at each session and was able to facilitate the administration of a depression symptom screening with the PHQ-9. Upon being placed in an examination room, patients were provided with paper copies of the screening tool by a member of the clinic staff and were asked to complete them privately. A cover sheet reinforced patients’ independent completion of the survey and maintained their confidentiality. Completed surveys were reviewed by the patient’s rheumatologist at the time of the visit. The social worker was later able to review results and follow up with patients by telephone as needed. This latter process served as a double check for any patients with positive screens for severe depression and/or suicidality who may have been overlooked. After an initial trial period, a meeting was held with clinic nursing staff and clinic front desk staff to discuss ideal workflow and potential barriers. From that discussion, two flowsheets were generated detailing how PHQ-9 screening would fit into clinic workflow for front desk personnel, nurses, and providers (Fig. 2), and indicating the steps a provider would take based on depressive symptom severity on the PHQ-9 (Fig. 3). One major change was to allow for nursing staff to provide screenings to patients, thereby decreasing our reliance on the social worker who could not be expected to be present at every clinic session.

The third intervention incorporated the PHQ-9 screening project into an existing pre-visit planning (PVP) process. As part of this process, QI informatics staff in the hospital sent out weekly lists of all SLE patients scheduled for an upcoming visit. A member of the QI team would then review medical records of all patients on the list who would be eligible for PHQ-9 screening at the time of their visit. A pre-existing PHQ-9 flowsheet in the EHR with dates and scores of all previous screenings helped to streamline this process. After determining which patients were due for screening, the information was collated with information from other projects and sent to all clinic staff as a single weekly “pre-visit planning list.”

The final intervention focused on increasing awareness of the project and of general mental health issues for patients and their families. A handout was prepared by our QI team and approved by the hospital. This handout included data on the association between mental health and SLE as well as resources for patients and families dealing with mental health issues.

### Study of the intervention(s)

Results of the interventions were reviewed and shared with the QI team on a monthly basis. Updated control charts were shared periodically with the larger Rheumatology division.

### Measures

Reports of PHQ-9 screening rates for a given month were sent out to the QI team at the beginning of the following month. All eligible patients seen in both the “Lupus Clinic” and general Rheumatology clinic were included in that month’s denominator. The numerator included all patients seen in a given month who had at least one documented PHQ-9 score in the EHR within the previous 12 months. The outcome of interest was the percentage of eligible patients who were seen in Rheumatology clinic in a given month who had been formally screened for depression with a PHQ-9 either at that month’s visit or within the 12 months prior to that visit. Secondary outcomes included the number of patients meeting criteria for different severity levels of depression as well as number of individuals for whom a mental health referral was placed in the EHR. We also measured the number of patients who ever provided a positive response when asked about endorsing thoughts of being “better off dead or hurting yourself” in the two weeks prior to the screening. Finally, we used the EHR Problem List and Medication List to calculate the number of patients with a documented behavioral health disorder (depression, anxiety, etc.) or history of antidepressant use, respectively.

### Analysis

**Fig. 4:**
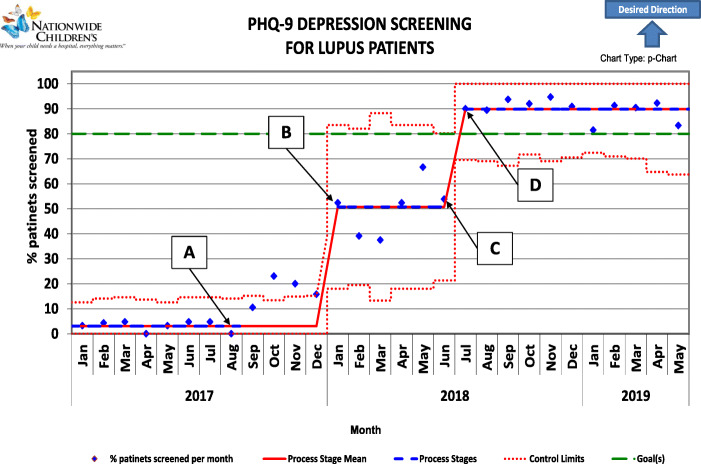
Annotated control chart. A. Project introduced at Rheumatology staff meeting. B. Initial PHQ-9 screens administered in clinic by social worker. C. New clinic workflow implemented. Pre-visit planning initiated. D. Patient forms updated to allow for easier tracking

Statistical process control, including the p-chart demonstrated in Fig. 4, was employed to monitor data throughout the course of the project. We followed the rules from the American Society for Quality (ASQ) to detect special cause variation as follows: (1) a single point outside the control limits, (2) two out of three successive points on the same side of the centerline and > 2 standard deviations from it, (3) four out of five successive points are on the same side of the centerline and farther than 1 standard deviation from it, and (4) a run of eight in a row are on the same side of the centerline.

### Ethical considerations

This QI project aimed to increase rates of formal depression screening among children and adolescents with SLE. The outlined process would allow for providers to detect patients with issues such as suicidal ideation which would require immediate action. It was important that providers not only be educated on the importance of screening but also on the appropriate steps to take in the case of a “positive” screen. Such steps were reviewed with our clinical psychologist, who was a member of the QI team, and outlined clearly on provider flowsheets.

## Results

### Rates of annual formal depression screening increased

Prior to introduction of this project in August 2017, monthly rates of documented formal depression screening among patients with c-SLE seen in the Rheumatology clinic at NCH averaged about 3.3 % per month. Over the course of the project about 20 patients with c-SLE were seen in the clinic per month. Over that period, two significant “center line shifts”, or nonrandom changes, in monthly screening rates were detected. By July 2018, at least 80 % of eligible patients seen in a given month were up to date with screening by the end of that month. That rate was sustained every month thereafter until May 2019 when data analysis was performed. The average screening rate over the last 11 months after the second center line shift was 89.2 %.

### Prevalence of depression in c-SLE was demonstrated

In the 22 months following the introduction of this project, 62 individuals were eligible for screening (Table 1). The demographics of patients screened were reflective of the c-SLE population in the NCH Rheumatology clinic. By the end of the project, 16 (25.8 %) individuals had a documented behavioral health disorder in the EHR while 11 (17.7 %) had a documented history of current or prior antidepressant use (Table 2). Fifty-six (90.3 %) eligible individuals were screened at least once over the course of the project. Twenty-two (39.3 %) of those individuals demonstrated at least mild depressive symptoms on their first PHQ-9 screening while 27 (48.2 %) demonstrated such symptoms on at least one screening over the course of the project. Seven individuals (12.5 %) endorsed having thoughts that they would “be better off dead” or of hurting themselves in at least one screening over the course of the project, requiring urgent suicide risk assessment, safety planning, and referral for mental health services. Nine individuals (16.1 %) were referred to NCH Psychology as a result of their PHQ-9 screening.

**Table 1:** Demographics and Baseline Mental Health Data

	N = 62
**Female, n (%)**	53 (85.5)
**Mean age at diagnosis, years (SD)**	13.32 (3.04)
**Race, n (%)**	
White	30 (48.4)
Black or African American	23 (37.1)
Asian	3 (4.8)
Other	6 (9.6)
**Insurance type, n (%)**	
Public	38 (65.0)
Private	21 (35.0)
**Lupus manifestations, n (%)**	
Nephritis	23 (37.1)
Discoid/Cutaneous Lupus	8 (12.9)
Neuropsychiatric SLE	3 (4.8)
Amplified or chronic pain	5 (8.1)
**Behavioral health history, n (%)**	
Prior antidepressant use	11 (17.7)
Documented history of behavioral health disorder	16 (25.8)

**Table 2:** PHQ-9 Screening and Referral Data

	N = 56
**Results of First PHQ-9 Screen, n (%)**	
Minimal	34 (60.7)
Mild	6 (10.7)
Moderate	14 (25.0)
Moderately Severe	1 (1.8)
Severe	1 (1.8)
History of at least one positive screen**	27 (48.2)
History of At Least One “Yes” Response to Question #9** *	7 (12.50 %)
**Referred to NCH Psychology**	9 (16.1)

*Maximum score is 27. Level of depression corresponds to the following scores: 0–4 = Minimal; 5–9 = Mild; 10–15 = Moderate; 15–19 = Moderately Severe; 20–27 = Severe.^13^

**Positive screen defined as mild depression or greater

**Question #9: Over the last 2 weeks, how often have you been bothered by any of the following problems? Thoughts that you would be better off dead or hurting yourself?

## Discussion

In successfully completing this project, we demonstrated the feasibility of implementing QI methodology to increase and sustain rates of formal depression screening among individuals with c-SLE in a Pediatric Rheumatology clinic. Such efforts were crucial in identifying the presence of depressive symptoms within our patient population, ensuring patient linkage to mental health supports, and normalizing the discussion around mental health.

We also demonstrated the prevalence of depression and suicidal ideation in individuals with childhood-onset SLE. Such results highlight the importance of formally screening for depression in patients with c-SLE. They also demonstrate the potential benefits that can be yielded for patients by identifying a clinical problem and approaching it from a stepwise approach with a multidisciplinary team.

To address the low rates of screening among our providers, we first determined common barriers to screening which included limited awareness of depression rates among c-SLE patients, limited time and resources during a clinic encounter, and lack of familiarity with how to respond to positive depression screenings. We addressed these barriers by educating providers on the rates of depression in children and adolescents with c-SLE as well as informing them about the available resources for mental health referrals in our institution, including referral to a clinical psychologist who was dedicated to Rheumatology. We then developed and posted a referral flowsheet for physicians to reference when reviewing PHQ-9 results in the clinic (Fig. 3).

We addressed the issue of clinic workflow by performing a nominal number of screenings in the clinic and assessing the effect on patient flow. We then worked with clinic staff to develop and incorporate a process that would minimally affect the clinic routine. A detailed worksheet indicating which patients were due for screening during their visit was placed in the staff workroom for easy reference throughout the week. Incorporating this pre-visit planning list into the daily routine of the clinic allowed staff to become familiar with the screening process and to appropriately prepare and administer the survey, ultimately minimizing the impact upon clinic work flow.

Various challenges were encountered and addressed using PDSA cycles. A major challenge to this project was ensuring that any individuals with a “positive screen” were detected and referred quickly and appropriately. An initial concern was that a screen would be positive for suicidal ideation, for example, but would not be reviewed by a provider until the patient had left the NCH campus. Providers were educated on how to review scores and the appropriate actions to take in the event of positive screens. This information was readily available for review in the clinic workroom in the form of an algorithm (Fig. 3). Scores were also later reviewed by our social worker who could follow up with patients over the telephone as needed.

Another challenge was ensuring that results of screenings reflected the feelings of the patient without external influence from the parent or guardian. To optimize the fidelity and confidentiality of the screenings, patients were given verbal instructions to complete the screening independently. A cover sheet highlighted this request and served as a way for results to be kept private. Patients and families were made aware of the mental health issues commonly observed with c-SLE through discussions with the physician, social worker, and psychologist. A handout outlining the mental health issues associated with c-SLE was distributed to patients and families to facilitate this conversation. No patients or families refused screening over the course of the study.

Pre-visit planning was initially a time-intensive process that required members of the QI team to navigate the EHR for multiple patients each week. However, this process was streamlined and grew more efficient as dedicated team members became more experienced in navigating the EHR and as more patients became up to date with annual screenings. In clinics without a dedicated social worker, any member of the clinical care or administration team could be trained to perform such weekly screenings ahead of time. Time invested up front will serve to save time during individual patient encounters.

To summarize, many obstacles were faced as we sought to achieve and sustain high rates of formal depression screening among children with c-SLE. With each new obstacle came an opportunity to refine our process so as to continue moving towards our goal. Measuring our progress on a monthly basis provided motivation as we were able to see the benefits of our work in real time. Of course, changes made to increase monthly screening rates could potentially have had unintended consequences such as taking away time that a provider could have spent addressing other aspects of lupus care. Overall time spent with a given patient could have also increased as a result of our interventions. While we did not formally track these balancing measures, we did discuss them frequently with clinic staff and providers.

## Limitations

There are a few factors which limit the utility and reproducibility of this study. First, the project was limited to English speaking patients in order to streamline the screening and review process. While this did not exclude many patients in our population, it could conceivably limit reproducibility in a practice with a large non-English speaking population. The PHQ-9 is widely available in multiple languages. Future steps could include determining how to incorporate multilingual screening tools into our workflow while ensuring that patients who use these tools are able to receive appropriate care where necessary. Reproducibility may also be limited by the availability of mental health resources at a given institution. For institutions without established mental health clinicians associated with their Rheumatology practice, providers could consider use of a standardized suicide risk assessment tool intended for implementation by nurses or physicians within medical settings, such as the Ask Suicide-Screening Questions (ASQ) Toolkit.[16] Providers might also wish to aggregate a list of local mental health providers in their area. Cultivating relationships with these community providers might allow for expedited referral when indicated. Patients may also be referred to their insurance plan to assist with locating local “in-network” mental health providers.

Finally, results of this study may be limited by the close monitoring that was performed by the dedicated QI team. Some practices, especially those at smaller centers, may not have access to such a team. A similar study performed at a small center might elucidate challenges that we did not address and allow for further determination of best methods to increase depression screening rates across various institutions. Further, our own high screening rates may have been reached and maintained in part due to providers knowing that their rates were being evaluated on a monthly basis. At the same time, keeping mental health at the forefront of provider’s minds was one goal of this project. It will be interesting to see if screening rates persist as we move further from the initial steps of this project.

## Conclusions

Depression is a major issue among children with chronic diseases but often goes undetected for years. Over the course of two years, we used QI techniques to systematically increase the rates of formal depression screening among providers who care for patients with c-SLE. The development of a standardized workflow which included pre-visit planning was crucial to the success of this project. As a result of our efforts, we increased the percentage of patients with c-SLE who received annual depression screening with the PHQ-9 from a monthly average of approximately 3.3 % to over 80 %. Not only did we increase screening rates, but we also were able to normalize the conversation around mental health among providers and families. Education of clinic staff, providers, patients, and their families was key to the success of such a program. Making use of an EHR for weekly pre-visit planning kept clinic staff cognizant of mental health screening and afforded them the opportunity to plan ahead. While access to mental health professionals and preexisting PHQ-9 flowsheets in the EHR may vary by institution, our hope is that readers of this paper will feel motivated to consider what resources they do have and then systematically develop a way to incorporate depression screening into their daily practice. Not only will this result in increased detection of depression in patients with c-SLE but it could also lead to improved subjective and objective clinical outcomes in a large portion of that patient population.

Future steps include broadening the patient population to include all rheumatic diseases as well as working to automate the pre-visit planning process using the EHR. Assessing for anxiety could also be explored. Finally, it would be worthwhile to track the outcomes of patients referred to mental health services in terms of mental health, lupus disease activity, medication adherence, and use of health care resources.

## Data Availability

The datasets used and/or analysed during the current study are available from the corresponding author on reasonable request.

## Abbreviations

AAP: American Academy of Pediatrics
CFF: Cystic Fibrosis Foundation
c-SLE: childhood-onset systemic lupus erythematosus
ECFS: European Cystic Fibrosis Society
EHR: electronic health record
NCH: Nationwide Children’s Hospital
PHQ-9: Patient Health Questionnaire-9
QI: quality improvement
SLE: systemic lupus erythematosus
USPTSF: United States Preventive Services Task Force
