# Correction to: Targeted provider education and pre-visit planning increase rates of formal depression screening in childhood-onset SLE

**DOI:** 10.1186/s12969-021-00631-0

**Published:** 2021-08-23

**Authors:** Evan Mulvihill, Rebecca Furru, Alana Goldstein-Leever, Kyla Driest, Stephanie Lemle, Darby MacDonald, Emily Frost, Vidya Sivaraman

**Affiliations:** 1 Department of Pediatrics, Divisions of a Rheumatology, Nationwide Children’s Hospital and The Ohio State University, Columbus, OH USA; 2 Department of Social Work, Nationwide Children’s Hospital, The Ohio State University, Columbus, OH USA; 3 Department of Pediatric Psychology and Neuropsychology, Nationwide Children’s Hospital and The Ohio State University, Columbus, OH USA; 4 Department of QI Services, Nationwide Children’s Hospital and The Ohio State University, Columbus, OH USA; 5 Department of Pediatrics, Division of Rheumatology, Nemours/A.I. duPont Hospital for Children, and Thomas Jefferson University, Wilmington, DE USA; 6 Department of Pediatrics, Division of Rheumatology, Nemours Hospital for Children Division of Rheumatology, 1600 Rockland Road, Wilmington, DE 19803 USA

**Correction to:** ***Pediatr Rheumatol*** **19, 116 (2021)**

**https://doi.org/10.1186/s12969-021-00576-4**

Following publication of the original article [1], the authors identified an error in the author name of Alana Goldstein-Leever.

- The incorrect author name is: Alanna Goldstein-Leever
- The correct author name is: Alana Goldstein-Leever

The author group has been updated above and the original article [1] has been corrected.
